# The SARS-CoV-2 Spike Protein Activates the Epidermal Growth Factor Receptor-Mediated Signaling

**DOI:** 10.3390/vaccines11040768

**Published:** 2023-03-30

**Authors:** Abdul Rasheed Palakkott, Aysha Alneyadi, Khalid Muhammad, Ali Hussein Eid, Khaled M. A. Amiri, Mohammed Akli Ayoub, Rabah Iratni

**Affiliations:** 1 Department of Biology, College of Science, United Arab Emirates University, Al Ain P.O. Box 15551, United Arab Emirates; 2 Department of Basic Medical Sciences, College of Medicine, QU Health, Qatar University, Doha P.O. Box 2713, Qatar; 3 Khalifa Center for Biotechnology and Genetic Engineering, United Arab Emirates University, Al Ain P.O. Box 15551, United Arab Emirates

**Keywords:** SARS-CoV-2, COVID-19, Spike, EGFR, hijacking, cancer, signaling

## Abstract

The coronavirus disease-19 (COVID-19) pandemic is caused by the novel severe acute respiratory syndrome coronavirus 2 (SARS-CoV-2). At the molecular and cellular levels, the SARS-CoV-2 uses its envelope glycoprotein, the spike S protein, to infect the target cells in the lungs via binding with their transmembrane receptor, the angiotensin-converting enzyme 2 (ACE2). Here, we wanted to investigate if other molecular targets and pathways may be used by SARS-CoV-2. We investigated the possibility of the spike 1 S protein and its receptor-binding domain (RBD) to target the epidermal growth factor receptor (EGFR) and its downstream signaling pathway in vitro using the lung cancer cell line (A549 cells). Protein expression and phosphorylation were examined upon cell treatment with the recombinant full spike 1 S protein or RBD. We demonstrate for the first time the activation of EGFR by the Spike 1 protein associated with the phosphorylation of the canonical Extracellular signal-regulated kinase1/2 (ERK1/2) and AKT kinases and an increase in survivin expression controlling the survival pathway. Our study suggests the putative implication of EGFR and its related signaling pathways in SARS-CoV-2 infectivity and COVID-19 pathology. This may open new perspectives in the treatment of COVID-19 patients by targeting EGFR.

## 1. Introduction

The new COVID-19 disease was identified for the first time in December 2019 in the province of Wuhan in China and it is caused by a new member of the coronavirus family called SARS-CoV-2 [1,2,3]. According to the latest statistics, over 111 million COVID-19 cases were reported causing around 2.5 million deaths worldwide (https://www.who.int/emergencies/diseases/novel-coronavirus-2019, accessed on 15 February 2023).

The molecular and cellular basis of the SARS-CoV-2 infection implicates the renin-angiotensin system (RAS) and more importantly angiotensin-converting enzyme 2 (ACE2) [3,4,5,6,7,8,9]. ACE2 is a host membrane-bound metallopeptidase with the catalytic site oriented extracellularly and is mostly expressed in the lung, heart, kidney, brain, and gut. In contrast to ACE which converts angiotensin I to the active vasoconstrictor, angiotensin II (AngII), ACE2 breaks down AngII to angiotensin-(1-7) [1,2,3,4,5,6,7,8,9] which are potent vasodilators and considered as negative regulators of RAS [10]. The implication of ACE2 in COVID-19 is thought to occur mostly in the very early stages of the viral infection and COVID-19 pathology. Indeed, the SARS-CoV-2-S protein, the spike glycoprotein (protein S), on the virion surface has been reported to bind the extracellular domain of ACE2 which is used as a co-receptor for target cell recognition and membrane fusion during the infection process [3,4,5,6,7,8]. ACE2 constitutes the main entry gate for another coronavirus, SARS-CoV [7]. In addition, in vivo studies showed a nice correlation between COVID-19 infection and the relative expression of ACE2 (positive) and its activity (negative). Furthermore, the receptor-binding domain (RBD) in the SARS-CoV-2 S protein has been identified and shown to bind strongly to human and bat ACE2 receptors [8]. The purified human recombinant RBD showed potent competitive action on the binding and, hence, the attachment of SARS-CoV-2 RBD to ACE2-expressing cells and their infection by the pseudovirus [3,8]. Thus, the RBD constitutes the most antigenic entity of the S protein and is used for the development of vaccines to prevent SARS-CoV-2 infection. Several spike-protein and RBD-based vaccines are in clinical trials [11].

The disease severity and mortality of COVID-19 have been reported to be increased in patients suffering from other chronic diseases such as cancer, diabetes, hypertension and cardiovascular problems. This was further evidenced in the patients who have been treated with anti-hypertensive drugs such as ACE inhibitors (ACEIs) and angiotensin receptor blockers (ARBs) [9,12,13,14,15,16,17,18]. In parallel, studies have shown that ACEIs and ARBs resulted in an upregulation of ACE2, favoring the entry and replication of the virus [18]. By contrast, SARS-CoV-2 by targeting ACE2 on the target cells causes down-regulation and inactivation of the latter. This downregulation generates an imbalance in favor of an over-accumulation of AngII, a potent vasoconstrictor. Such an effect was shown to increase oxidative damage which leads to inflammation and pulmonary fibrosis [19]. Moreover, inflammation, cytokine storm, and thrombosis associated with pulmonary injury constitute other important clinical features of COVID-19 pathology. This suggests the implication of other molecular actors such as the protease thrombin via its proteinase-activated receptors (PARs), the purinergic receptors, cytokine receptors, and lipid mediators. Thus, the inhibition of these pathways has been proposed as a promising therapeutic approach to prevent thrombotic and inflammatory processes during COVID-19 pathology [20,21].

Although a large body of evidence using in vitro studies and in silico data strongly supports the thesis that ACE2 is necessary for SARS-CoV-2 entry, still, we cannot rule out that additional factors and/or alternative cell surface receptors may be also implicated in SARS-CoV-2 entry [22]. Among these possible receptors, the G protein-coupled receptors (GPCRs) and receptor tyrosine kinase (RTKs) constitute valid candidates based on their tissue abundance and pivotal roles in human and animal physiology. Indeed, several previous studies reported the hijacking of GPCRs and RTKs and their function by various pathogens during pathogenesis. This includes microbial pathogens such as bacteria and viruses such as the SARS-CoV and the adrenergic receptor and the epidermal growth factor receptor (EGFR) as the targets [23,24,25]. Thus, we hypothesize that during SARS-CoV-2 infectivity, the virus may also use EGFR expressed on the epithelial lung cells as the receptor/co-receptor target for its entry.

Here, we have examined the effect of the full-length SARS-CoV-2 and DRB Spike 1 protein on the activation of EGFR and its related downstream signaling pathways consisting of AKT and ERK1/2 phosphorylation in ACE2 expressing lung (A549). As a cellular model, we used cancer cell lines known for their expression of EGFR and differential expression of ACE-2 including ACE-2 expressing lung (A549) and colon (HT-29) cancer cell and ACE-2 non-expressing Cervix (HeLa) adenocarcinoma cells.

## 2. Materials and Methods

### 2.1. Cell Culture, Chemicals and Antibodies

All cell lines used in this study were obtained from Cell Line Service (CLS)-GmbH. HT-29 and HeLa cells were maintained in Dulbecco’s modified eagle medium (DMEM) (Cat. # 03640, Gibco, Life Technologies, Rockville, UK). A549 cells were maintained in RPMI (Cat. # 00506 Gibco, Life Technologies, Rockville, UK). All media were complemented with 10% fetal bovine serum (FBS) (Cat. # 02187 Gibco, Life Technologies, Rockville, UK) and 100 U/mL penicillin streptomycin glutamine (Cat. # 01574 Gibco, Life Technologies, Rockville, UK). AG1478 (Cat. # 141438) was purchased from Abcam, Cambridge, UK. Antibodies against phospho-EGFR (Cat. # 4407), EGFR (Cat. # 4267), phospho-ERK1/2 (Cat. # 9106), ERK1/2 (Cat. # 4695), phospho-AKT (Cat. # 9271), AKT (Cat. # 9272), Survivin (Cat. # 2803), ACE2 (Cat. # 4355), anti-mouse (Cat. # 7076), and anti-rabbit (Cat. # 7074) were purchased from Cell Signaling, Technology, Danvers, MA, USA. SARS-CoV-2 spike protein 1 (Cat. # DAGC091) was purchased from Creative Diagnostics, Shirley, NY, USA. SARS-CoV-2 spike RBD protein (Cat. # 40592-V08B) was purchased from Sino Biological, Beijing China. Human EGF Recombinant Protein (Cat. # PHG0313) was purchased from Gibco, Life Technologies, Rockville, UK.

### 2.2. Whole Cell Extract and Western Blotting Analysis

Cells (0.5 × 10^6^) were seeded per well in a 60 mm culture dish and cultured for 24 h before treatment. After treatment with or without EGF, spike 1 protein or Spike RBD protein, cells were washed twice with ice-cold PBS, scraped, pelleted and lysed in RIPA buffer (Pierce) supplemented with protease inhibitor cocktail (Roche) and phosphatase inhibitor (Roche). After incubation for 30 min on ice, cell lysates were centrifuged at 14,000 rpm for 20 min at 4 °C. Protein concentration of lysates was determined by BCA protein assay kit (Thermo Scientific, Rockford, IL, USA) and the lysates were adjusted with lysis buffer. Aliquots of 25 µg of total cell lysate were resolved onto 6–15% SDS-PAGE. Proteins were transferred to nitrocellulose membranes (Thermo Scientific, Rockford, IL, USA) and blocked for 1 h at room temperature with 5% non-fat dry milk in TBST (TBS and 0.05% Tween 20). Incubation with specific primary antibodies was performed in blocking buffer overnight at 4 °C and this was true for all the primary protein antibodies used in this study. Notice that all the lysates were freshly loaded when different phospho-proteins were analyzed. However, the same membrane of the phospho-protein was stripped to examine the corresponding total protein loaded except for pERK1/2 where the proteins were always freshly loaded due to stronger anti-pERK1/2 binding. Moreover, a β-actin blot was also used in parallel to double-check that similar amounts of proteins were loaded in every gel’s lane. Horseradish peroxidase-conjugated anti-IgG was used as a secondary antibody. Immunoreactive bands were detected by ECL chemiluminescent substrate (Thermo Scientific, Rockford, IL, USA) and chemiluminescence was detected using the LiCOR C-DiGit blot scanner and Image Studio Lite 5.0 Software. Densitometry quantification of the Western blots was carried out using ImageJ software.

## 3. Results

### 3.1. Spike and RBD Activate EGFR, AKT, and ERK1/2 in A549 Cells

**Figure 1:**
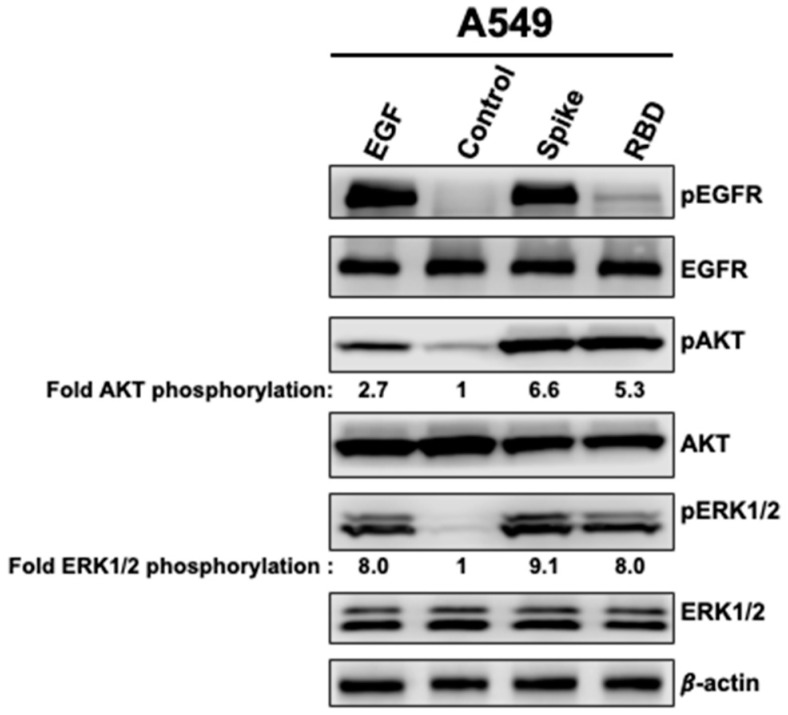
Effect of SARS-CoV-2 Spike 1 protein on epidermal growth factor receptor (EGFR), Ak strain transforming (AKT) and extracellular signal-regulated kinase 1/2 (ERK1/2) activation. A549 cells were treated or not with EGF (5 μg/mL) for 15 min or with full-length Spike 1 (2.5 μg/mL) or with the spike receptor-binding domain (RBD) (5 μg/mL) protein for 5 min then whole-cell extracts were subjected to western blot analysis for phosphoEGFR (pEGFR), phosphoAKT (pAKT), phosphor ERK1/2 (pERK1/2) and their respective total levels.

First, we examined the effect of Spike 1 and RBD on the phosphorylation status of EGFR and its related kinases, AKT and ERK1/2, in ACE2-expressing lung cancer cells (A549). A549 cells were treated with EGF (5 μg/mL) for 15 min or with either whole Spike protein (2.5 μg/mL) or spike RBD (5 μg/mL) for 5 min. Cells were then washed with ice-cold PBS and lysed as described in materials and methods. The treatment of cells for 5 min with Spike (2.5 μg/mL) promoted a strong phosphorylation of EGFR, AKT, and ERK1/2, which was either similar (EGFR and ERK1/2) or even higher (AKT) to that promoted by stimulation with the maximal dose of EGF (5 μg/mL) (Figure 1). On the other hand, RBD (5 μg/mL) under a similar condition showed almost no effect on EGFR phosphorylation while it significantly induced AKT and ERK1/2 phosphorylation (Figure 1). This result suggests the specific targeting of EGFR by the full spike 1 protein of SARS-CoV-2 but not by its RBD. Additionally, our data suggest that RBD-mediated AKT and ERK1/2 activation is independent of EGFR activation and that other molecular targets and/or intracellular mechanisms may be involved.

**Figure 2:**
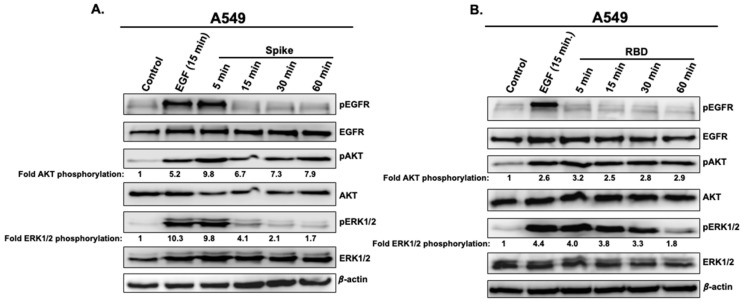
Time-course accumulation of pEGFR and pERK1/2 in A549 cells treated with full length Spike 1 protein and its RBD. Cells were treated or not with 2.5 μg/mL full length Spike protein (**A**) or 5 μg/mL RBD (**B**) at different times as indicated (5, 15, 30 and 60 min) and the protein levels of pEGFR and pERK1/2 and their respective total levels were determined by western blot. The treatment with EGF (5 μg/mL) was carried out for 15 min.

We next performed a time-course analysis with both Spike 1 and RBD on the phosphorylation status of EGFR and ERK1/2. For this, A549 cells were treated with Spike 1 (2.5 μg/mL) (Figure 2A) or RBD (5 μg/mL) (Figure 2B) for 5, 15, 30, and 60 min. Stimulation with EGF (5 μg/mL) as a positive control was carried out for 15 min. As shown in Figure 2A, Spike 1 induced a rapid and transient phosphorylation of EGFR and ERK1/2 with a peak at 5 min of stimulation and a sharp decrease after 15 min. Spike 1-mediated AKT activation, on the other hand, was also observed upon 5 min of stimulation but this was more sustained in time (Figure 2A). For RBD, there was no clear EGFR phosphorylation induced regardless of the stimulation time (Figure 2B). This is consistent with our observation in Figure 1. However, RBD promoted rapid and strong activation of both ERK1/2 and AKT that remained persistent even after 60 min of stimulation (Figure 2B). The quantification of the different blots indicates that both Spike 1 and RBD promoted protein phosphorylation to comparable levels as that mediated by EGF (Figure 2A,B).

Together, these data further confirm the specific activation of EGFR by Spike 1, but not RBD, but they also demonstrate the implication of additional and/or different mechanisms in AKT and ERK1/2 activation whether cells were activated by Spike 1 or RBD.

### 3.2. Activation of AKT by Spike 1 and RBD Is EGFR-Dependent

**Figure 3:**
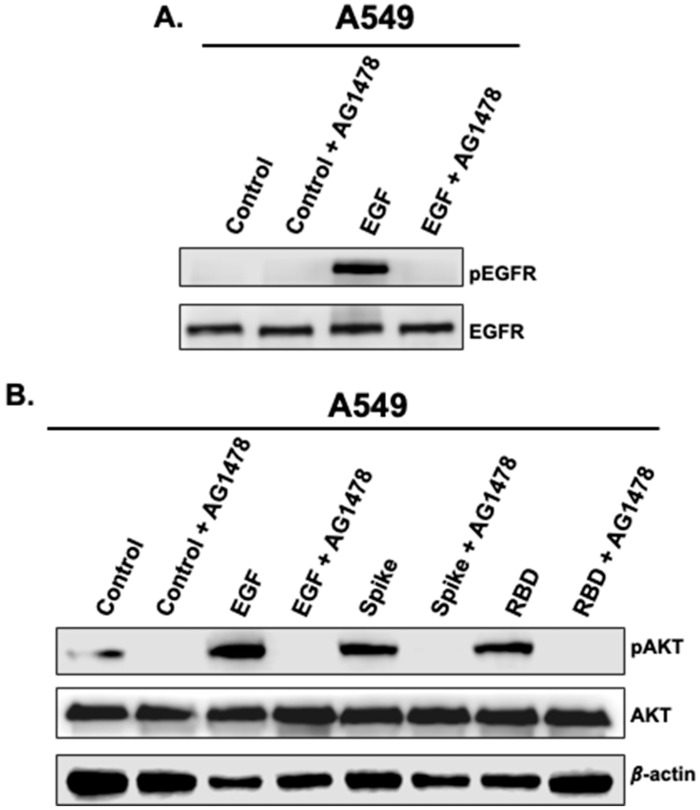
Blockade of EGFR inhibits SARS-CoV-2 spike 1-mediated activation of AKT in A549 cells. A549 were first pre-treated with AG1478 (10 μM) for 15 min prior to treatment with EGF (**A**,**B**), full-length spike 1 protein or spike RBD protein (**B**) as described in Figure 1. Whole cell extracts were resolved on SDS-PAGE and protein levels were determined by western blot.

Next, we decided to explore the targeting of EGFR and its related downstream AKT survival pathway by Spike 1 and RBD by using the selective EGFR antagonist, AG1478. As shown in Figure 3A, the pre-treatment of A549 cells with AG1478 (10 μM) fully blocked EGFR phosphorylation induced by stimulation with EGF (5 μg/mL) indicating the specificity of the response. We found that AG1478 completely abolished AKT phosphorylation induced by EGF, Spike 1, or RBD (Figure 3B). This finding strongly suggests that Spike 1- and RBD-mediated AKT phosphorylation is dependent on EGFR activation.

### 3.3. The Activation of AKT and ERK1/2 by Spike 1 and RBD Occurs in ACE2-Expressing Cells

Next, we wanted to examine the relationship between the expression of ACE2 as the main entry receptor for SARS-CoV-2 and the targeting of EGFR and its downstream signaling pathways. For this, we extended our study to two different cancer cell lines known for their differential expression of ACE2, the highly ACE2-expressing (HT-29 colon cancer) and ACE2 non-expressing (HeLa cervix adenocarcinoma) cells. We first confirmed such a statement by investigating the endogenous expression of ACE2 in the different cancer cells. As shown in Supplementary Figure S1, while ACE2 was expressed in A549 and HT-29 cells, no detectable expression of ACE2 was observed in HeLa cells. It is noteworthy to mention that HT-29 cells expressed a high level of ACE2 (~five fold compared to A549 cells).

**Figure 4:**
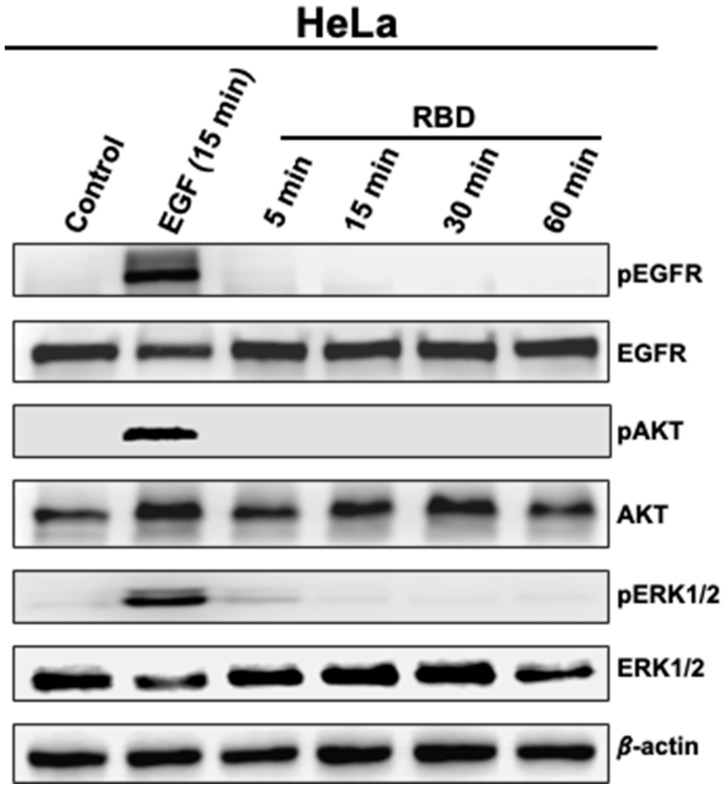
Time-course accumulation of pEGFR, pAKT, and pERK1/2 in RBD-treated HeLa cells. HeLa cells were treated or not with 5 μg/mL RBD protein for the indicated time points (5, 15, 30 and 60 min), and the protein levels of pEGFR, pAKT and pERK1/2, and their respective total levels were determined by western blot. As a positive control, the treatment with EGF (5 μg/mL) was carried out for 15 min.

Next, we investigated the effect of RBD on the phosphorylation status of EGFR, AKT, and ERK1/2 in HeLa and HT-29 cells. For this, HeLa (Figure 4) and HT-29 (Supplementary Figure S2) cells were treated with RBD (5 μg/mL) for 5, 15, 30, and 60 min using the stimulation for 15 min with EGF (5 μg/mL) as a positive control. Interestingly, in HeLa cells lacking ACE2, neither AKT nor ERK1/2 was phosphorylated upon RBD treatment regardless of the stimulation time (Figure 4). By contrast, EGF stimulation induced AKT and ERK1/2 phosphorylation in HeLa cells (Figure 4). In HT-29 cells, RBD did not promote any EGFR phosphorylation, unlike EGF (Supplementary Figure S2) and this was consistent with our observations in A549 cells shown in Figure 2B. Interestingly, RBD induced a rapid and sustained AKT and ERK1/2 phosphorylation (>5 and >2 fold, respectively) in HT-29 cells (Supplementary Figure S2) similar to ACE2-expressing A549 cells (Figure 2B). Together with our observation on endogenous ACE expression shown in Supplementary Figure S1, these data confirm that RBD-mediated AKT and ERK1/2 phosphorylation is independent of EGFR activation as shown in Figure 2, and strongly suggest the implication of ACE2 in such pathways.

### 3.4. Effects of Spike 1 and RBD on the Cell Survival Marker, Survivin

**Figure 5:**
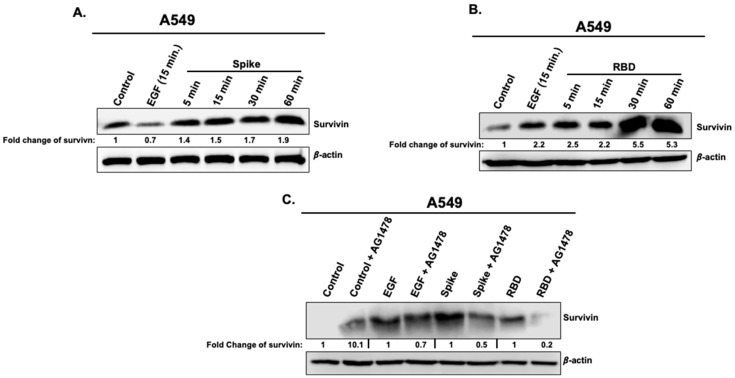
SARS-CoV-2 full length spike 1 protein and its RBD promote survivin expression in A549 cells. A549 cells were treated or not with EGF (5 μg/mL) (**A**,**B**), full-length spike 1 protein (2.5 μg/mL) (**A**) or spike RBD protein (5 μg/mL) (**B**) at different times (5, 15, 30 and 60 min) as indicated and the protein level of survivin was determined by Western blot. (**C**) A549 cells were first pre-treated or not with AG1478 (10 μM) for 15 min prior to treatment with EGF (5 μg/mL) or spike RBD protein (5 μg/mL) and the protein level of survivin was determined as described above.

Since EGFR, AKT, and ERK1/2 pathways are known for their role in cell proliferation and cell survival, we wanted to link our data on the phosphorylation status of these proteins especially EGFR and AKT with the induction of the anti-apoptotic protein, survivin, which belongs to the inhibitor of apoptosis (IAP) family and considered as the key marker for the activation of the survival pathway in cancer cells. Moreover, the activation of survivin was shown to be induced by both AKT and ERK1/2 signaling in cancer cells. For this, A549 cells were treated with either Spike 1 (2.5 μg/mL) (Figure 5A) or RBD (5 μg/mL) (Figure 5B) for 5, 15, 30, and 60 min using stimulation for 15 min with EGF (5 μg/mL) as a positive control. Both Spike 1 (Figure 5A) and RBD (Figure 5B) induced the increase in survivin expression in a time-dependent manner with a maximal response at 30 and 60 min. Furthermore, similar to Spike 1- and RBD-mediated AKT phosphorylation, both Spike 1- and RBD-mediated survivin activation in A549 cells was also significantly diminished (~ by 2 to 5 fold) by the EGFR antagonist, AG1478, demonstrating the implication of EGFR/AKT axis (Figure 5C) in the upregulation of survivin. It is noteworthy to mention that, the pretreatment with AG1478 also increased the expression of survivin as compared to cells that were not pretreated with AG1478 (Figure 5C). Such an increase was observed even in the non-stimulated (control) cells (Figure 5C). These observations demonstrate that Spike 1- and RBD-promoted EGFR/AKT pathway in A549 cells is associated with the activation of survivin which may elicit the survival of the cancer cells.

## 4. Discussion

In this study, we report for the first time the possible hijacking of EGFR and its related downstream signaling pathways by SARS-CoV-2 Spike 1 protein and its RBD in lung cancer cells (A549). We demonstrated that Spike 1-induced AKT activation occurred in an EGFR-dependent manner since it was drastically blocked by AG1478. On the other hand, we found that Spike 1 and RBD also elicited the activation of the survival pathway in A549 cells. Indeed, both Spike 1 and RBD induced the expression and the activation of the anti-apoptotic protein, survivin, which belongs to the inhibitor of apoptosis (IAP) family and is considered a the key marker for the activation of the survival pathway in cancer cells. Such a response was very consistent with the phosphorylation of AKT in these cancer cells. Such a response was very consistent with the phosphorylation of AKT in these cancer cells. This may constitute a solid molecular and cellular rationale to explain the increased risk of infectivity by SARS-CoV-2 and its severity in cancer patients as recently reported by several groups [26,27].

Regarding the implication of our findings in the pathophysiology of COVID-19, recent studies showed that cancer patients were more vulnerable to the SAR-COV-2 infection. Although COVID-19 was reported to have a low death rate of ~2% in the general population, patients with cancer and COVID-19, have at least a three-fold increase in the death rate. Dai et al. showed that patients with lung cancer, gastrointestinal cancer, or breast cancer had the highest frequency of critical symptoms including the highest death rates [27]. Patients with lung cancer and gastrointestinal cancer had a death rate of 18.18% and 7.69%, respectively [27]. Interestingly, they showed that cancer patients that received targeted therapy that includes the EGFR-tyrosine kinase inhibitors showed the lowest death rate compared to cancer patients who received immunotherapy, chemotherapy, or surgery [27]. Another study carried out at the Gustave Roussy Cancer Centre (France) by Albiges et al., showed that 27% of the cancer patients with COVID-19 developed clinical worsening and 17.4% died [26]. Here, we showed that SARS-CoV-2 activates the EGFR and its downstream signaling pathways controlling cell survival and proliferation. We also found that the inhibition of EGFR abolished the SARS-CoV-2 activation of AKT.

**Figure 6:**
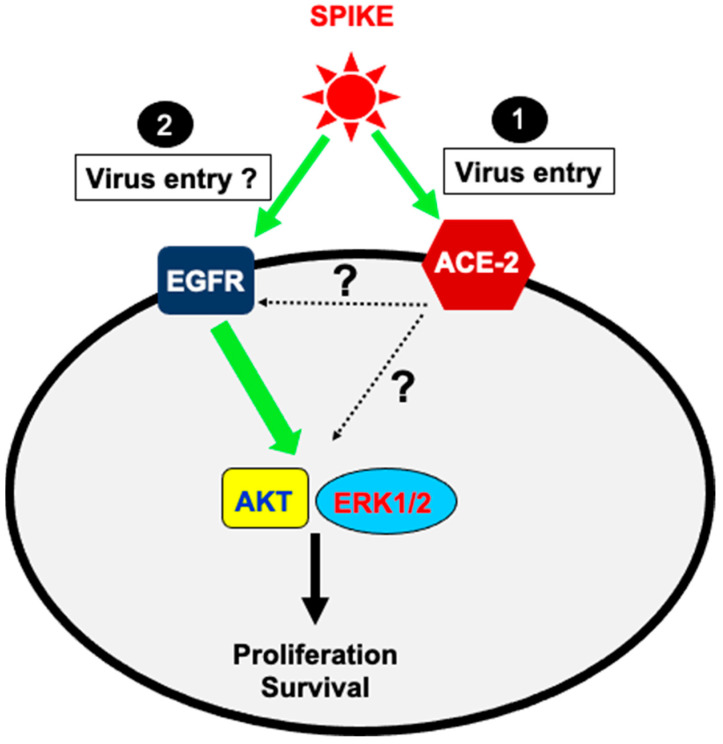
Speculative model for the targeting of EGFR and its downstream signaling by SARS-CoV-2 spike 1 protein.

At the molecular level, our in vitro data provide, for the first time, evidence that SARS-CoV-2 Spike 1 protein activates EGFR and its downstream signaling pathways, AKT and ERK1/2. This is very consistent with the well-established concept of the hijacking of cell surface receptors and their activity/signaling by pathogens including viruses [28,29,30] and bacteria [25,31]. This implies that pathogens use GPCRs and RTKs at the cell surface of the target cells during the infection process leading to their entry into the target cells. Interestingly, previous studies also showed the role of EGFR and its downstream signaling pathways in viruses/bacteria pathogenicity [24,25] being consistent with our findings on SARS-CoV-2 spike protein. Indeed, EGFR was shown to be important during influenza infection [24]. In addition, similarly to our data, the Salmonella Rck membrane protein has been reported to bind and activate EGFR and its mediated signaling resulting in receptor/bacteria co-internalization and cell infection [25]. This also occurs with GPCRs since both viruses and bacteria were demonstrated to bind and activate GPCRs resulting in the co-internalization of viruses [30,31] or bacteria [31] with the target receptors. As stated above, we showed that the Spike 1-induced AKT activation occurred in an EGFR-dependent manner in A549 cells since it was blocked by EGFR blockade (AG1478). This supports the conclusion that the activation of the AKT/survival axis by Spike 1 depends on EGFR activation (Figure 6). Moreover, the differential effects of Spike 1 and RBD suggests a different mode of activation and/or molecular pathways involved. One possible explanation is that Spike 1 may directly target EGFR while RBD uses other targets at the cancer cell surface including ACE2 resulting in AKT and ERK1/2 phosphorylation independently of EGFR (Figure 6). Furthermore, our time-course analysis shown in Figure 2 indicated interesting differences in the phosphorylation kinetics in A549 cells when compared EGFR, AKT, and ERK1/2, on one side and Spike 1 versus RBD on the other. Indeed, Spike 1 mediated EGFR and ERK1/2 phosphorylation in a transient manner since it was observed within 5 min of stimulation and declined after. This was different with EGF inducing the phosphorylation at 15 min of stimulation. However, AKT phosphorylation was more sustained and remained significantly high even after 60 min of stimulation. The effect of RBD on AKT and ERK1/2 phosphorylation was more or less similar to Spike 1. However, and surprisingly, RBD did not promote any EGFR phosphorylation. This suggests the implication of other EGFR-independent pathways with or without the implication of ACE2. This aspect requires further investigation.

Overall, our data are consistent with direct targeting of the EGFR/AKT pathway, but this does not rule out the alternative pathway consisting of the implication of the canonical ACE2 pathway via transactivation of EGFR at the cell surface or intracellular crosstalk between their intracellular pathways (Figure 6). Of course, further studies are required to demonstrate whether Spike protein directly binds to EGFR or not and what would be the implication of the canonical ACE2 pathway. Even though we did not investigate all the aspects of EGFR-dependent pathways and SARS-CoV-2 infectivity, we believe that our data will pave the way for further investigation of the exact role of EGFR in SARS-CoV-2 infection and pathogenicity. More interestingly, our study using cancer cells reveals a possible molecular basis for COVID-19′s complications and severity observed in cancer patients by considering EGFR as a potential target for the management of such pathological and clinical situations.

In conclusion, our data are consistent with Spike 1-mediated EGFR activation in an ACE2-dependent manner, which may be directly via binding to EGFR (1) or indirectly (2) via ACE2 crosstalk, as shown in the hypothetic model in Figure 6. This results in the activation of AKT and survivin pathway in both EGFR- and ACE2-dependent manner as well as ERK1/2 pathway. Such spike 1 protein-engaged signaling pathways play a role in cancer cell survival that may explain the increased risk of COVID-19 infectivity reported in cancer patients.

## Data Availability

All data are available in the manuscript and in the supplementary material.

## Supplementary Materials

The following supporting information can be downloaded at: https://www.mdpi.com/article/10.3390/vaccines11040768/s1, Figure S1: Analysis of the expression of the endogenous ACE2 in cance cell lines; Figure S2: Time-course accumulation of pEGFR, pAKT, and pERK1/2 in RBD-treated HT-29 cells.
