# Supplementary material for: The SARS-CoV-2 Spike Protein Activates the Epidermal Growth Factor Receptor-Mediated Signaling
Source: Vaccines (Basel). 2023 Mar 30;11(4):768. doi: 10.3390/vaccines11040768 (PMC10141239; doi:10.3390/vaccines11040768)
Supplement: Supplementary file 1 [file vaccines-11-00768-s001.zip › vaccines-2271894-SI.pdf]

Supplementary materials

The SARS-CoV-2 Spike Protein Activates the Epidermal Growth Factor Receptor-Mediated Signaling

Abdulrasheed Palakkott [1,#], Aysha Alneyadi [1,#], Khalid Muhammad [1], Ali H. Eid [3], Khaled, Amiri [1,2], Mohammed Akli Ayoub[1,q,*] and Rabah Iratni [1,*]

[1] Department of Biology, College of Science, United Arab Emirates University, Al Ain PO Box 15551, UAE.
[2] Khalifa Center for Biotechnology and Genetic Engineering, United Arab Emirates University, Al Ain PO Box 15551, UAE.
[3] Department of Basic Medical Sciences, College of Medicine, QU Health, Qatar University, Doha, Qatar
[#] The authors contributed equally to the work.
[q] Present address: Biology Department, College of Arts and Sciences, Khalifa University, P O Box 127788, Abu Dhabi, UAE
* Correspondence: Rabah Iratni (R_iratni@uaeu.ac.ae), Mohammed Akli Ayoub (mohammed.ayoub@ku.ac.ae)

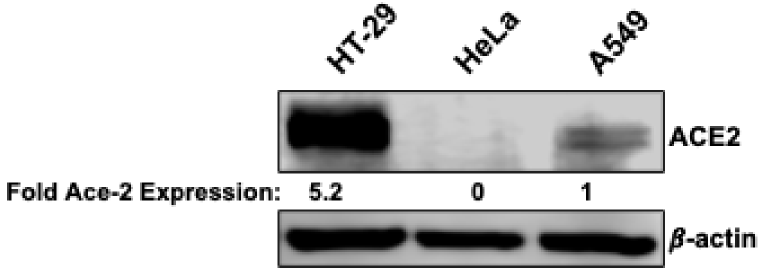

Figure S1. Analysis of the expression of the endogenous ACE2 in cance cell lines. A549, HT-29, and HeLa cells were used for the endogenous expression of ACE2 using SDS-PAGE followed by western blot using anti-ACE2 antibody.

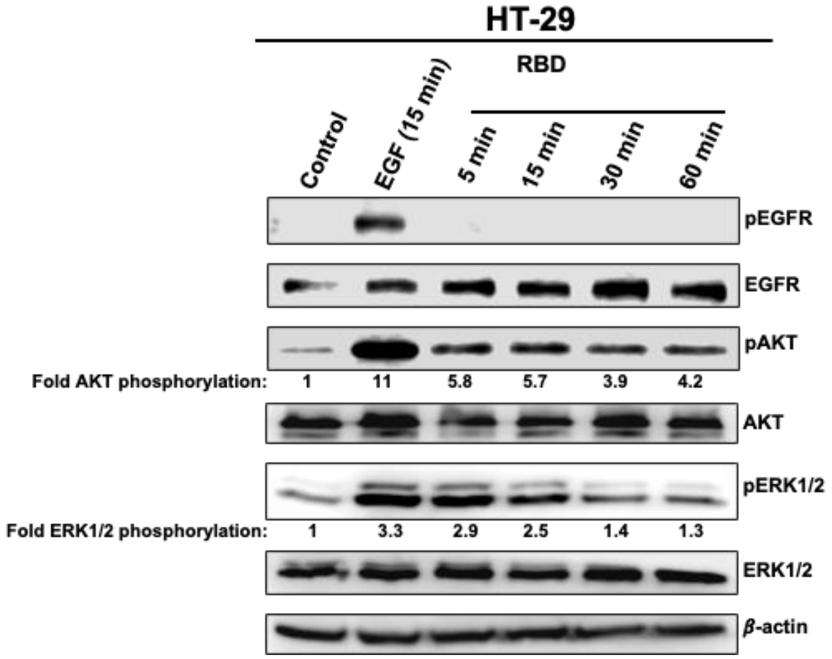

Figure S2. Time-course accumulation of pEGFR, pAKT, and pERK1/2 in RBD-treated HT-29 cells. HT-29 cells were treated or not with 5 µg/mL RBD protein for the indicated time points (5, 15, 30 and 60 min), and the protein levels of pEGFR, pAKT and pERK1/2, and their respective total levels were determined by western blot. As a positive control, the treatment with EGF (5 µg/mL) was carried out for 15 min.
